# Supplementary material for: R2R - software to speed the depiction of aesthetic consensus RNA secondary structures
Source: BMC Bioinformatics. 2011 Jan 4;12:3. doi: 10.1186/1471-2105-12-3 (PMC3023696; doi:10.1186/1471-2105-12-3)

crcB

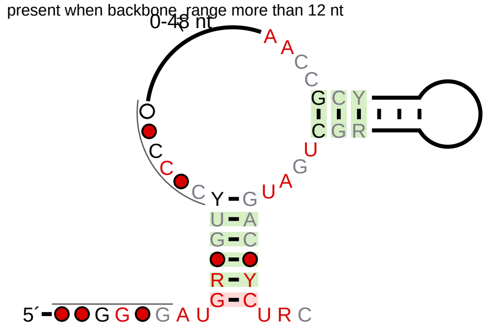

crcB NC_008578.1/805741-806202

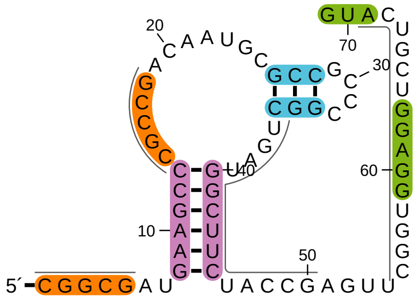

crcB NC_008578.1/805741-806202 alt-struct=2

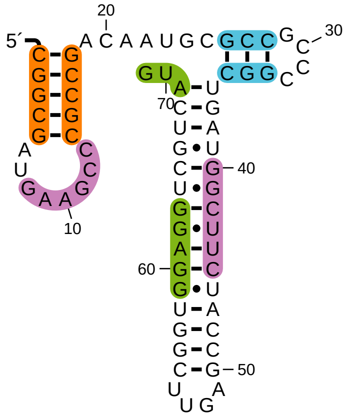

crcB NZ_ABYJ01000311.1/4968-4495

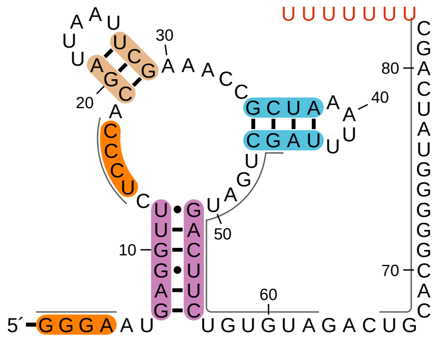

crcB NZ_ABYJ01000311.1/4968-4495 alt-struct=2

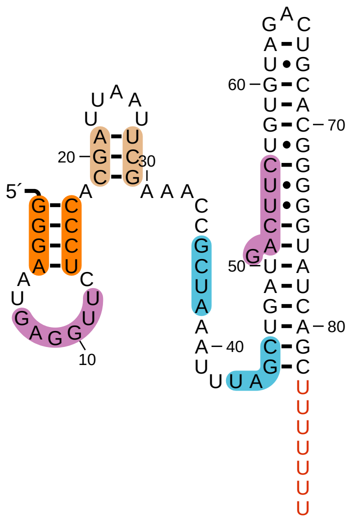

crcB NZ_ABYJ01000311.1/4968-4495 no-shading=1

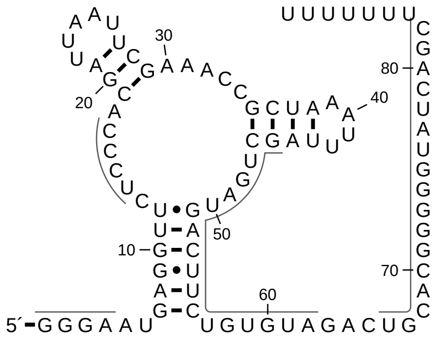

crcB NZ_ABYJ01000311.1/4968-4495 no-shading=1 anyangle=1

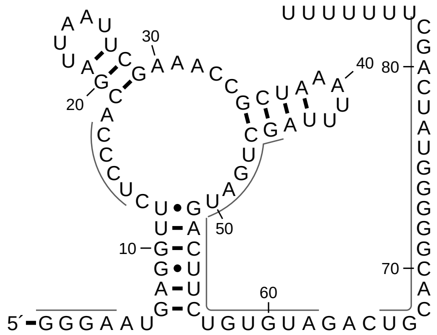

crcB NZ_ABYJ01000311.1/4968-4495 no-shading=1 anyangle=1 drawcirc=1

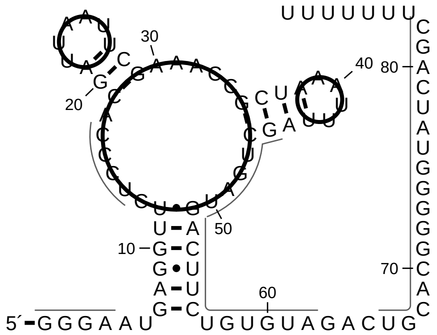

crcB NZ_ABYJ01000311.1/4968-4495 no-shading=1 anyangle=1 drawedges=1

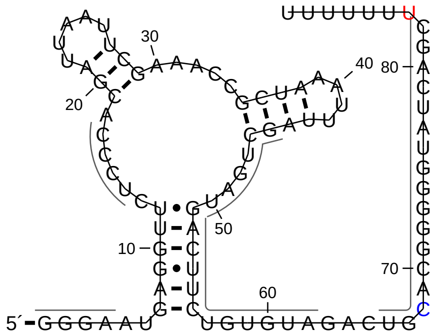

crcB R2R-paper=1

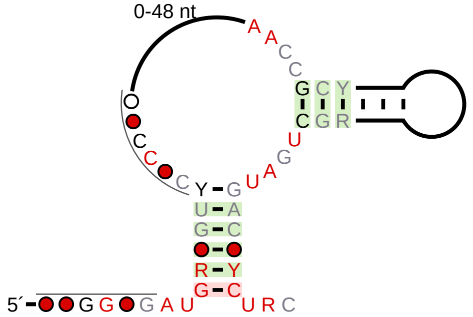

crcB skeleton-with-bp

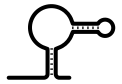

crcB-P2
subfam_weight=0.308697

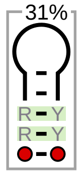

crcB-pknot1
subfam_weight=1

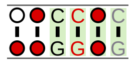

Supplement: Additional file 1 — Source code and example input and output files. C++ and Perl source code is provided. Installation and usage instructions are given in the user manual (Additional file 2), which is also a part of this archive. Example input files for R2R are included in the "demo" subdirectory of this archive, and R2R's raw output on these examples in PDF and SVG format is supplied in the "demo/output-pdf" and "demo/output-svg" subdirectories. Files can be retrieved from the tgz archive file using programs such as WinZip (Windows), StuffIt Expander (Mac) or the tar/gzip commands (UNIX). [file 1471-2105-12-3-S1.TGZ › R2R-1.0.1/demo/output-pdf/crcB.pdf]
